# Visual Pathways Serving Motion Detection in the Mammalian Brain

**DOI:** 10.3390/s100403218

**Published:** 2010-04-01

**Authors:** Alice Rokszin, Zita Márkus, Gábor Braunitzer, Antal Berényi, György Benedek, Attila Nagy

**Affiliations:** Department of Physiology, Faculty of Medicine, University of Szeged, H-6720 Szeged, Hungary; E-Mails: alice@phys.szote.u-szeged.hu (A.R.); markus@phys.szote.u-szeged.hu (Z.M.); bgabor@phys.szote.u-szeged.hu (G.B.); tony@phys.szote.u-szeged.hu (A.B.); benedek@phys.szote.u-szeged.hu (G.B.)

**Keywords:** dorsal stream, ventral stream, ascending tectofugal system, caudate nucleus, posterior thalamus, motion detection

## Abstract

Motion perception is the process through which one gathers information on the dynamic visual world, in terms of the speed and movement direction of its elements. Motion sensation takes place from the retinal light sensitive elements, through the visual thalamus, the primary and higher visual cortices. In the present review we aim to focus on the extrageniculo-extrastriate cortical and subcortical visual structures of the feline and macaque brain and discuss their functional role in visual motion perception. Special attention is paid to the ascending tectofugal system that may serve for detection of the visual environment during self-motion.

## 1. Introduction

Visual motion perception is the process through which humans and other animals orient themselves to their own movements and those of the objects comprising their environment, *via* light-transmitted signals processed by their visual system.

Motion perception is one of the most important capabilities of the visual system. Changes in the environment usually provide important information for the animal. Irrespectively of whether it is a predator or a prey, such information is crucial for survival. Beside the detection of light and dark, perception of motion seems to be the oldest and most important feature of the visual system. Despite the fact that a wide range of visual animals lack binocular or color vision, the visual perception of motion seems to be a general property that can be difficult to substitute [1].

The three-dimensional dynamic world is projected on the surface of the retina as a two-dimensional spatio-temporal pattern of light intensity. From this picture the visual system has to reconstruct the changes in the visual field, and it also has to make a distinction between ground and figure, shape, form and extent, that is the whole three-dimensional structure [2]. For this reconstruction the detected motion is crucial as well. Furthermore, the visually detected motion is important for the monitoring of self-motion [3].

From the beginning of the research into vision, where and how these procedures happen in the brain was a key question [4,5]. In the striate visual system, only some aspects of the motion information are processed [1,6–9], but it is more and more obvious that without the extrastriate cortical and subcortical structures the whole processing cannot be accomplished [10–12]. During recent years, as more and more information has come to light concerning vision, the role and characteristics of these extrastriate structures have become a focus of the attention of neuroscientists.

It is established that there are different parallel loops, which consist of the extrastriate cortices (cortical regions surrounding the suprasylvian sulcus in the feline brain, the middle temporal area (MT), the medial superior temporal area (MST), the superior temporal polysensory area (STP) in the primate brain) and subcortical structures (pretectum, accessory optic system, basal ganglia, thalamus). According to the classical theory, these extrastriate structures play subservient and complementary roles in motion sensation. The two-stage motion processing theory is a generally accepted hypothesis [13–16]. It assumes that at the first stage the analysis of the object features as one-dimensional components occurs in early visual areas, depending on orientation-selective mechanisms sensitive to the motion of individual component contours. The second stage elements are regarded as pattern motion detectors, and these are possibly higher extrastriate cortical areas integrating the output of the first stage analyzers to construct the actual direction of the coherent pattern. However, more and more evidence is found to raise the suspicion that the two-stage theory might be incomplete for modeling the visual motion analysis [10,11,17]. Rather, the extrastriate structures have equal and coordinate functions in receiving direct input from the lower, primary stages of the visual stream, not only through indirect connections from the primary visual cortical areas.

The aim of the present review is to give a detailed description of the extrastriate visual structures of the feline and the macaque brain and discuss their functional role in visual motion perception. Special attention was paid in the second part of this review to the ascending tectofugal system in the feline brain that may serve the perception of self-motion.

## 2. Retino-Geniculo-Cortical Visual Pathways in Primates

**Figure 1.**
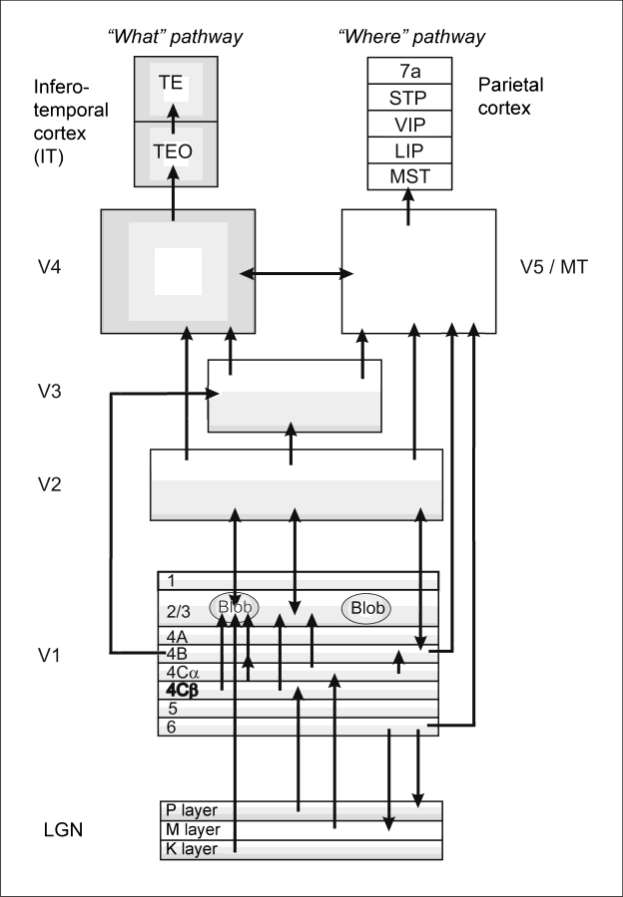
Schematic representation of the primate visual pathways. The left half of the figure represents the ventral (“what”) stream, while the right side shows the hierarchical organization of the dorsal (“where”) stream. Abbreviations: LGN—Lateral geniculate nucleus, V1, V2, V3, V4—Primary (first), second, third, fourth visual cortices, respectively, TEO—Posterior inferior temporal cortex, TE—Anterior inferior temporal cortex, V5/MT—Middle temporal area (fifth visual cortex), MST—Medial superior temporal area, LIP—Lateral intraparietal area, VIP—Ventral intraparietal area, STP—Superior temporal polysensory area, 7a—Visual area 7a in the parietal cortex (Brodmann’s terminology).

In the visual pathways of vertebrates, motion perception spreads from retinal cells to higher cortical areas (Figure 1).

The first stage is the retina, comprising of three functional layers: rods and cones, bipolar cells, and ganglion cells. The horizontal cells between rods and cones and the amacrine cells between ganglion cells establish lateral connections. Morphologically, 10 layers may be distinguished, the description of which, however, we set aside as being outside the scope of the present study. The first integrative stage in the processing of an image is the layer of ganglion cells. To our present knowledge, Three major types of ganglion cells might be distinguished, although at least 17 types are known altogether [18]. Two of the major types are quite well characterized; these are the magnocellular-projecting (M) and parvocellular-projecting (P) cells. The third type, the koniocellular-projecting (K) cells [19], only relatively lately became a focus of attention and their function is as yet poorly understood. The studies of Kuffler [20] pointed out that retinal ganglion cells depict the visual space in a concentric ‘on’ and ‘off’ manner. Although these studies were not carried out in primates, since then it has been established that the same principle stands for primates as well [21]. An ‘on’ zone is defined as the part of the receptive field, which upon stimulation with a suddenly appearing light stimulus, evokes an excitatory response (spike train) in the given cell. An ‘off’ zone evokes the same response upon the disappearance of that stimulus. The direction- and motion-sensitivity of retinal cells were first described by Barlow and Levick [22] registering the electrical activity of ganglion cells extracellularly.

Pathways originating in retinal ganglion cells project on the lateral geniculate nucleus (LGN) of the diencephalon, traditionally considered to be part of the thalamus, while applying more strict anatomical criteria it is part of the metathalamus [23]. As discussed later, in connection with tectal pathways, retinal ganglion cells also project upon the superficial layers of the superior colliculus (SC, optic tectum in lower vertebrates) as monosynaptic afferents. The lateral geniculate body of primates consists of six layers. The concentric on/off receptive field arrangement is to be found here as well. That these cells should be directionally sensitive is quite unlikely, however, they may serve as input to higher order cells exhibiting that sort of sensitivity. Which LGN cells have a role in movement detection remains an open question. The four dorsal layers comprise the neurons of the parvocellular system. These neurons are color-sensitive, while they exhibit no special sensitivity to luminance modulation, that is, their contrast-sensitivity is low. The remaining two layers belong to the magnocellular system. Cells here respond to a wide spectrum of light, and they exhibit no color opponency. Cells receiving wide-spectrum chromatic input are highly contrast sensitive [24]. Isoluminant chromatic stimuli have but little effect on motion sensation [25], which does not support the role of the parvocellular layers in motion perception. Figure/background segmentation in these layers is poor too [26]. Therefore, the magnocellular cells of LGN layers 5 and 6 are more likely candidates. These cells primarily respond to transient stimuli.

In primates, LGN projects upon V1, the primary visual cortex. Parvocellular and magnocellular systems bifurcate in V1, where (in primates) directional sensitivity first appears, in the cells of cortical layer 4C [27]. From here, motion information is transmitted toward the dorsal stream, *via* layers 4B and 6. Directionally sensitive cells have been described in layer 3 as well. These cells have a small receptive field, and they exhibit strong end-inhibitory capability, which makes them optimal candidates for multiple visual functions. Livingstone and Hubel [28] considered the directionally selective cells of V1 to be the basic units of motion perception. V1 layers 4B and 6 project directly upon the temporal cortex in a monosynaptic manner [29,30]. On the other hand, V1 layer 4B also projects to the temporal cortex indirectly, *via* cortical area V3 [31,32]. Allman and Kaas [33] were the first to describe this rather small, cytoarchitectonically distinct, myelin-rich projection area at the dorsal bank of the superior temporal sulcus of the northern owl monkey (Aotus trivirgatus). This extrastriate area representing the contralateral visual hemifield based on single-unit registrations was named middle temporal area (MT).

Zeki [34] described an analogous area in the macaque brain, which was given the name V5 (V5 and MT, therefore, are used interchangeably to refer to this area.) MT receives its major projections from the striate cortex (V1) and V2. It gives out an efferentation of thick myelinated fibers, which refers to rapid visual processing [35]. Zeki was the first to describe that V5 neurons are especially sensitive to motion. Many cells responded vividly to stimuli (bright spots or dark/bright lines) moving in a given direction, while they failed to respond to movement in the opposite direction. The shape of the stimulus did not seem to matter, until the direction was optimal. Other cells required that the stimuli should move with a certain orientation in a given direction. These cells were like V1 complex cells, however, they had significantly larger receptive field and they were directionally selective. V5 cells of identical directional selectivity exhibited a tendency of columnary organization. Only a few V5 cells showed wavelength-sensitivity [36–39]. Behavioral experiments following cortical lesions corroborated that MT is an area participating in motion perception. Newsome *et al*. [40] found specific oculomotor deficits related to the matching of pursuit movements to the speed of the target after minor cortical lesion. This corroborates the hypothesis that pursuit movements require speed information [41], which is transmitted by MT. This is further supported by MT efferents to cerebellar vermis *via* the pontine dorsolateral nuclei. Both areas play a role in the organization of pursuit movements, and their cell discharge rates are modulated by target speed and the speed of eye movements [42,43]. Movshon and colleagues [44] showed that responding to a grating complex; MT cells are sensitive to the real direction of movement, not to the movement direction of the individual spatial Fourier-components. There are also MT-cells which are maximally sensitive to speed, regardless of spatial and temporal frequency [40]. Andersen *et al*. have also demonstrated that the area MT plays a fundamental role in the structure-from-motion perception, which means the perception of three-dimensional shape from motion cues. This mechanism is needed for the three-dimensional vision and provides the third dimension, depth, from two-dimensional cues, to the flat image developing on the retina [45,46].

The fact that V5 receives direct input from the highly directionally sensitive V1 4B [29] and the cytochrome-oxidase rich areas of V2 [47] throws light on the origins of the directional sensitivity of V5—so pronounced that V5 is often referred to as ‘movement area’. V5 is assumed to be responsible for visual motion perception and smooth pursuit movements. V5 lesions in humans and in the macaque monkey impair the ability of the animal to discriminate the movement direction of a random noise pattern. However, a few weeks after the lesion this function reappears, possibly meaning that other areas are capable of taking it over [48].

Perge and colleagues [49] described a center/surround organization of MT receptive fields. This makes MT especially sensitive to contrasts both in space and time. Recently appeared multivoxel studies, fMRI activation of MT corresponds to the motion perception of the observer, while this is not true for the rest of the visual areas [50]. However, a study of Zeki *et al*. [51] seems to contradict the concept of MT as a perceptive area. In that study, volunteers were shown dot patterns. The dot patterns were moved either in different directions, or in the same direction in front of the two eyes. As long as the dot patterns were moved in just the opposite directions, they knocked each other out perceptually, in spite of the fact that real movement occurred in front of the eyes. Activity registered over MT was characteristic of the physical movement itself, not its perception. Finally, in several areas of the monkey brain, e.g., in areas V4 and MT it was found that single unit activity depended on where within the receptive field the animal was focusing its attention [52–55].

The spatio-temporal selectivity of neurons in the MT was discovered by Lui *et al*. [56]. They recorded the neuronal responses in marmoset monkeys to high-contrast sine-wave grating stimulation, which revealed that the majority of neurons had band-pass spatial and temporal frequency tuning, and that the selectivity for these parameters was largely separable. Inseparable spatio-temporal tuning properties could only be detected in approximately one third of the neurons, in the typical form of an increased optimal temporal frequency as a function of increasing grating spatial frequency. However, most of these interactions have been found to be weak and only 10% of neurons exhibited spatial frequency-invariant representation of speed. Cells with an inseparable spatio-temporal tuning were most commonly located in the infragranular layers, raising the possibility that they form part of the feedback pathway from the MT to the caudal visual areas. Spatial frequency tuning curves were approximately scale-invariant on a logarithmic scale, however, temporal frequency tuning curves covering different parts of the spectrum demonstrated significant and systematic changes. According to these data, MT neurons can be regarded as similarly built spatial frequency filters, each covering a different dynamic range. The small proportion of speed-tuned neurons, together with the laminar position of these units, complies with the hypothesis that an explicit neural representation of speed emerges from computations performed in the MT.

The role of areas beyond MT in motion perception is not clear. MT/V5 sends especially strong projections to the medial superior temporal area, which supports the role of the latter area in motion perception [57,58]. Eifuku and Wurtz [59] pointed out that the medial superior temporal area in macaque monkey consists of a dorsomedial (MSTd) and a lateroventral (MSTl) part. According to the authors, MSTd processes optic flow information [60] as in, information on the own motion of the observer, while MSTl is specialized for external motion. Therefore, this area is capable of providing information on self-movement, the surrounding space and the shape of objects. Likewise, cells in the superior temporal sulcus (STS) process self-motion information. Cells at the upper bank specialize in dynamic information concerning the ongoing action, while cells at the lower bank are sensitive to static information related to posture. In humans and primates STS may be responsible for the recognition of complex visual stimuli, such as facial-, hand-, and body-movements. Beyond that, STS may have some role in multimodal sensory integration.

Cells in the parietal lobe are sensitive to motion too, though these cells seem to foster movement regulation, rather than perception [61–63]. The caudal part of the parietal lobe is responsible for the synchronized processing of somatic and visual information, therefore primarily serving the purpose of visually controlled action. In the intraparietal sulcus, five visual areas have been described, based on morphological and physiological criteria. These are: ventral intraparietal (VIP), medial intraparietal (MIP), posterior intraparietal (PIP), anterior intraparietal (AIP), and lateral intraparietal (LIP). Area 7a is located in the lateral parietal surface, areas 7 and V6A in the medial parietal wall. Single unit recordings have yielded both somatosensory and visual responses. It seems that VIP represents the perioral areas, while MIP the extrapersonal space. According to Sakata *et al*. [64,65], AIP is responsible for the visual guidance of hand movements and grabbing. LIP is basically the parietal eye field. It has been shown through single unit recordings that this area may be linked to both changes in gaze direction and the sensory stimulus inducing those changes. Motter and Mountcastle [66] described cells in 7a that were sensitive to radial changes of visual stimuli, like the ’shrinking’ of an image. This obviously means that this area is sensitive to changes in spatial proximity.

The three main cell types of the retina, the magnocellular-projecting, the parvocellular-projecting and the koniocellular-projecting retinal ganglion cells, and the pathways starting out from them may act as multiple movement sensitive systems working in parallel. Despite that little information is available about the function of the koniocellular system, it is suggested that all three systems may play a role in motion perception to different extent. Nassi and Callaway [32] provided evidence for the existence of a direct connection between the intermediate (koniocellular) layers of the LGN and the area MT/V5, based on which we can conclude that the koniocellular pathway may have a specific role in motion perception. Functionally speaking, visual systems of the brain serve multiple, more or less dissociable ends. For instance, for image segmentation or figure/background discrimination necessitates high level retinotopic organization, while the control of eye movements does not require such high resolution, as the system sums up visual information from the entire visual field [67]. Such distinctions support the possibility of a double motion perceptive system assumed to exist in the mammalian brain [68]. According to that theory, a cortical system is responsible for the analysis of movements occurring at particular points in the visual field, while an accessory system originating in the brain stem analyzes motion in the visual field as a whole. Though these systems are separate, there is interaction between them, and they also occur at different times during the ontogenesis [69,70]. Accessory systems of brain stem origin possibly play but little role in motion perception. However, beside eye movement control, ascending pathways originating in the tectum (in mammals the SC) might participate in providing feedback about head and body movements, thus, in the control of those movements.

Thomas Albright and his colleagues hypothesized that the system for motion perception combines image elements in space and time [71,72]. For instance, it is the system’s task to determine in what direction our hand moves, and it has to be able to tell that what moves is a hand. Based on positron emission tomography (PET) studies these tasks can be linked to the ventral and dorsal visual streams, which have been originally hypothesized to exist by Ungerleider and Mishkin [5]. The key idea is that beyond the primary visual cortex dissociated pathways serve the purposes of identification and localization. During the PET examinations the occipital cortex was activated in both ‘where’ and ‘what’ tasks, while, for example, the identification of faces selectively activated occipitotemporal areas, and spatial localization tasks selectively activated occipitoparietal ones.

## 3. The Ascending Tectofugal System in the Feline Brain

The existence of separate geniculate and extrageniculate visual systems in the feline brain has been proved in both morphological and physiological studies. Beside the lateral geniculate nucleus, altogether nine subcortical structures have been found that receive afferents directly from the retina [74]. Of these structures, the SC and the tectal pathway have attracted the most research interest during the past 25 years. In this chapter, we summarize the morphological and physiological properties of an ascending tectofugal pathway in the mammalian brain that seems to function exclusively *via* a tectal route without direct contribution from the geniculostriate route.

The story started in 1980, when Otto Creutzfeldt and Lennart Mucke attempted to record the visual properties of neurons in the claustrum *via* stereotaxic targeting. They were repeatedly able to record visually highly active neurons, but these turned out to be located outside the border of the caudal portion of the claustrum on histological control. This serendipitous finding led to the discovery of a novel visual area along the anterior ectosylvian sulcus (AES) [75] and initiated the experiments that we describe below. It should be noted that the existence of the anterior ectosylvian visual area (AEV) was simultaneously detected by Olson and Graybiel, who had similarly searched for visual activity in the claustrum [76,77]. Later, the extent of the visual region was extended to the cortex throughout almost the whole length of the AES, including its rostral gyral cortical region; that was called the insular visual area (IVA) [78–80] a name that was later found not to be totally appropriate [81]. The morphological experiments of ours and others confirmed the tectal source of visual information towards the AES cortex. Hence, this area now seems to be the only cortical visual area that is provided with visual afferentation entirely bypassing the lateral geniculate complex [74,80,82]. The AEV receives thalamic afferents mainly from the lateral medial-suprageniculate nuclear complex (LM-Sg), while a smaller fraction of the afferentation comes from the medial part of the nucleus lateralis posterior (LPm) [76]. The source of the cortical afferentation to the AEV is mainly the posterior-medial division of the lateral suprasylvian area (PMLS) [83,84]. The predominant targets of efferentation of the visual neurons along the AES are the LM-Sg and the intermediate and deep layers of the SC, although it sends visual efferents to the PMLS, to the frontal visual areas (lower bank of the cruciate sulcus and the lower lateral side of the frontal part of the feline brain), to the amygdala and other cortical and subcortical structures, outside of the lateral geniculate complex and the A17 region [83]. The substantial corticothalamic connections directed our attention to the LM-Sg. This nucleus of the posterior thalamus had earlier been paid less attention to in morphological and physiological analysis because of problems with the definition of its borders. Acetylcholinesterase staining offered a chance to circumscribe (and to locate) this thalamic area exactly [85]. Anatomical tracing experiments proved that there is a noteworthy convergence of inputs from a wide anteroposterior and mediolateral aspect of the intermediate and deep layers of the SC to the neurons in the LM-Sg [86]. In fluorescein double-staining experiments we observed, that collicular neurons send bifurcating axons towards the ipsilateral and also the contralateral LM-Sg [87]. Similar bifurcation of collicular axons were suggested in the primate, where the two SCs provide visual and oculomotor information for each frontal eye field [88]. Anatomical experiments revealed that both the substantia nigra (SN) and the pedunculopontine tegmental nucleus of the feline brain send efferents to the ventral part of the LM-Sg. An other substantial projection was traced from the ventral LM-Sg to the posterior dorsolateral part of the caudate nucleus (CN) [89]. This finding extended our observations to the CN and the SN, parts of the feline brain that are directly involved in visuomotor control. This is in line with the findings that the fastigial nuclei of the cerebellum were found to send bifurcating axons to the right and left LM-Sg and SC [90].

The summarized results showed that the ascending tectal axons carrying visual information constitute a fiber pathway linking the mesencephalon with the dorsal thalamus and then with a number of telencephalic centers. Nuclei of the mammalian posterior thalamus *i.e.*, the LM-Sg and the lateral posterior-pulvinar complex (LP-Pul), and their sauropsidian and avian homolog, the nucleus rotundus, occupy a central position in this pathway [91,92]. The neurons in this pathway exhibit unique physiological properties, which in no way resemble those described in the geniculostriate pathway. The same receptive field properties have been found in neurons along the AES including the IVA, the LM-Sg and later the posterior dorso-lateral part of the CN and the SN [93,94]. Hence, below we summarize our physiological findings, irrespective of the region in question. The most intriguing finding was the absolute absence of retinotopic organization, in contrast with the impressive retinotopy in the geniculostriate pathway [95]. Receptive fields consistently included the area centralis and extended practically over the entire visual field of the corresponding eye, not only in the AEV, but in all the regions found in this pathway. Others have raised the possibility that the neurons in the AEV are arranged according to their directional preference [96], although our observations do not support this concept [97]. The absence of traditional topographic coding raised the idea that there could be another type of spatial coding in this system. Indeed, Middlebrooks and coworkers described panoramic coding properties in the auditory neurons along the AES [98] and similarly we found evidence for panoramic coding of spatial visual information in the ascending tectofugal system [99]. The majority of the visual neurons proved to be selective for the stimulus location; they gave significantly different responses to stimuli from different spatial locations. These make one assume that the visual neurons of the ascending tectofugal system have similar abilities to serve as panoramic localizers [94,96]. The regions of maximal sensitivity within the visual field are widely distributed among the LM-Sg, the AEV and the CN neurons. Thus, populations of maximally active neurons can accurately code the spatial position of the visual information. This is a distributed population code of visual information that is based on panoramic localizer neurons.

**Figure 4.**
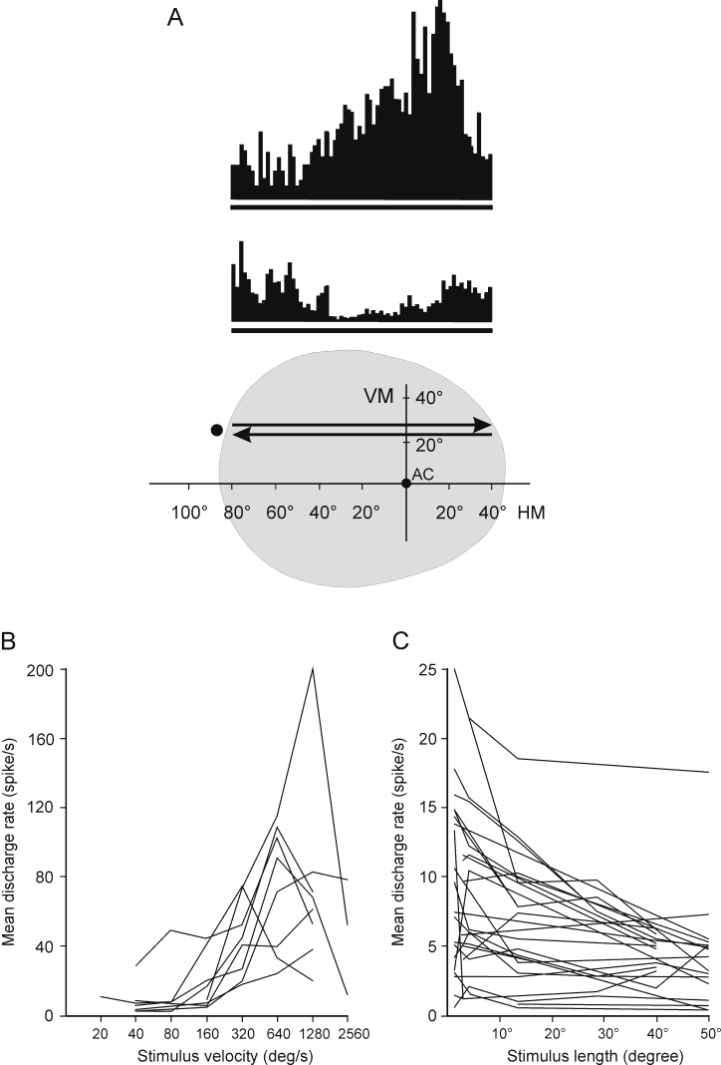
Visual properties of neurons in the ascending tectofugal system. A: Top: Peristimulus time histograms (PSTHs) of a directionally selective LM-Sg visual neuron in the ascending tectofugal system. Bottom: the position and movement of the stimulus in the visual field of the cat. The black spot left to the arrows symbolizes the moving visual stimulus. The upper and lower PSTHs correspond to the response of the neuron to the stimulus moving along the trace indicated by the upper and lower arrows, respectively. The grey part represents the extent of the visual receptive field. Abbreviations: AC: area centralis, HM: horizontal meridian, VM: vertical meridian. B: Velocity response curves for a spot stimulus (2° in diameter) of eight visual AEV neurons of this system. Note the high responsiveness of the units to high velocities! C: Effect of the length of the light stimulus (1° in width) on the response of visual neurons in the AEV. Note the maximal responsiveness of the neurons to small stimuli!

A striking physiological characteristic of these neurons (*i.e.*, in the AEV, IVA, LM-Sg, CN and SN) is their overwhelming sensitivity to movement in the receptive field. First, we found that the neurons were primarily sensitive to small stimuli moving very rapidly in a specific direction in the huge receptive field (Figure 4).

High directional sensitivity, together with the preference for a high stimulus speed was a characteristic that turned out to be similarly valid for the cells in the SC, which is evidently the main source of visual information for this pathway [100]. The neurons along the extrageniculate visual pathway seemed not to be sensitive to the orientation or shape of the stimuli. This supports our concept that the receptive field properties make these cells serve as “motion” or “novelty” detectors.

The visual information processing depends critically upon the integration of spatial and temporal information. The sinusoidally modulated grating is an elementary component of the visual scene in the sense that any two-dimensional visual object can be represented by an appropriate combination of these gratings [101,102]. Responses of neurons to drifting gratings of different spatial and temporal frequencies can be interpreted in terms of the dimensions and distribution of spatially and temporally summed excitatory and inhibitory components within their receptive fields [103,104]. Thus, the discussion of the spatiotemporal filter properties of neurons in the ascending tectofugal system may contribute to an understanding of the role of the system in visual information processing and the related sensory-motor actions. Similarly to the findings of the classical studies, the intermediate and deep layers of the SC (SCi, SCd), the LM-Sg, the AES cortex and the CN possessed very similar spatiotemporal spectral receptive field properties. The neurons responded optimally to low spatial frequencies and exhibited low spatial resolution and low-pass spatial tuning. The temporal frequency properties have been found to be similar in the different examined structures. Optimal responses were recorded to high temporal frequencies and the cells displayed high temporal frequency cut-off and narrow temporal frequency tuning. These findings indicate that these neurons act as effective spatio-temporal filters in the low spatial and high temporal frequency domain, which suggests that this system plays a major role in the detection of sudden changes in the environment and in the analysis of the velocity of movement. In human vision, the motion detectors cover a wide range of spatial frequencies, but do not seem to be relevant in terms of high spatial frequencies [105]. All motion detectors are apparently fine tuned for temporal and spatial frequencies [105], the narrow tuning aiding the velocity detection and the analysis of the object in motion, regarding shapes, edges, and so on [106,107]. Neurons responding optimally to low spatial and high temporal frequencies with a narrow tuning [108] have all the capacities to perceive the optic flow [109]. Thus, they could be optimal candidates for tasks involved in the perception of self-motion. The ascending tectofugal visual system may play a role in recording movements of the visual environment relative to the body, and thus it may participate in the adjustment of motor behavior in response to environmental challenges.

Other interesting aspect of the ascending tectofugal system is its sensitivity to several sensory modalities. Beside the visual neurons, auditory, somatosensory and multisensory neurons were also found. The visual and the somatosensory modalities predominated in the ascending tectofugal system. The sensory receptive fields were extremely large. The visual and auditory receptive fields covered the whole physically approachable sensory field, while the somatosensory receptive fields covered the whole body surface of the animal. The receptive field properties of the multisensory neurons were similar to those of the unimodal neurons. Similarly to the absence of retinotopic organization, we observed no signs somatotopic organization.

## 4. Lateral Suprasylvian Areas of the Feline Brain

Palmer *et al*. [110] in 1978 gave the first description of the cortical regions surrounding the suprasylvian sulcus and it is widely agreed that LS cortex consist of six visuotopically organized areas: anteromedial and anterolateral lateral suprasylvian area (AMLS and ALLS), posterior medial lateral suprasylvian (PMLS), posterior lateral lateral suprasylvian (PLLS), dorsal and ventral lateral suprasylvian areas (DLS and VLS).

These regions are connected to other cortical and subcortical structures which play major role in the processing of visual information, and the analysis of motion information. Because of the reason that the LS areas receive not only extrageniculo-extrastriate visual information originating from the SC but also geniculo-striate visual input [111,112] we discuss these cortical regions of the feline brain separated from the ascending tectofugal system. The collicular information is relayed through the pulvinar and the lateral posterior nucleus of the thalamus to the LS [113–115].

LS is classically associated with motion perception and are thought to play a major role in the analysis of motion. It is implicated in attention shifts [116–118], speed discrimination [119], the integration of complex motion [120] and the detection of forms that are in motion [121,122]. From the six areas which were originally described, the posteromedial lateral suprasylvian sulcus was most extensively studied.

Several studies have shown that the PMLS neurons are motion-sensitive, have large receptive fields that are exquisitely selective for the direction of motion and prefer relatively higher velocities (10–40°/s) [17,118–126]. Behavioral studies have also demonstrated the involvement of the PMLS cortex in motion processing [115,119,120,127–130]. This extrastriate area is traditionally thought to be one of the second stage analyzers, but studies with drifting plaid rarely found pattern motion-sensitive cells in the PMLS, most of the neurons were found to be component motion-sensitive, and it was suggested that in this area the response to the direction of motion is secondary to the determination of orientation, and that motion signals are integrated in other parts of the feline cerebral cortex [123]. There has been disagreement in the literature concerning the presence or absence of orientation selectivity in the PMLS [118,119,122,131–135]. However, Li *et al*. [17] found that the pattern motion or the component motion sensitivity may not be a fixed feature of a certain cell, but the direction tuning of the PMLS neuron can vary with the orientation element of the stimulus. When the component lines of the stimulus were much shorter than the size of the receptive field, the majority of cells were selective to the direction of pattern motion, while only a small population was sensitive to the direction of component motion. Response profiles of the majority of the neurons became more component-motion selective with the size increment of orientation element in the stimulus by elongating the component lines in the patterns. These results suggest that additionally to the widely discussed orientation-sensitive mechanism, certain types of other processes, relatively independent of the one-dimensional orientation cue, may also be involved in the determination of the motion’s direction, because such a dynamic variation of pattern motion and component motion sensitivity would probably require dual underlying mechanisms. The two mechanisms may act parallel in a dynamic competition, where one rises as the other falls, depending upon the strength of the orientation element in the stimulus. As a result, when one mechanism prevails over the other, it would respond like a pattern motion- or component motion-sensitive cell, otherwise unclassified. Even at relatively low levels of the visual system, some kind of non-orientation-based processing may coexist with the orientation-sensitive processing in dynamic competition. During other experiments [125] dealing with the optic flow analyzing feature of the PMLS it has been found that this area is not likely to be specialized for the analysis or discrimination of different flow patterns, but may play some kind of relay role in the optic flow information processing. On the other hand, Villeneuve *et al*. [136] investigated if the PMLS neurons can signal the direction of motion of complex random dot kinematograms (RDKs), wherein the composing elements do not provide any local coherent motion cues. According to their results the PMLS neurons can signal the direction of a complex RDK, which requires the integration of local motion over a large spatial area. The cells in this area are capable of binding local motion cues, even if these cues are separated by relatively large spatial displacement. These data suggest that most PMLS cells can signal the direction of motion of complex RDKs only when the latter stimulates the area beyond their classical receptive fields, presumably through intra- or inter-cortical (AMLS, PLLS, DLS) connections or by afferents from subcortical nuclei (LP-pulvinar complex) involved in complex motion analysis. The coding way in PMLS is likely to be coarse, rather than sparse, since the majority of the cells were direction-selective and almost always broadly tuned to the direction of motion, even for higher order stimuli such as complex RDKs, and the way of coding did not vary with the stimulus context. In this study they could not detect pattern-motion-sensitive cells, but they emphasised that the stimulus they used is so fundamentally different that any direct comparison between the plaid-defined and the random-line-defined pattern-motion can be excluded. These studies support the hypothesis that the PMLS is one of the most important early cortical stages in motion integration.

The posterolateral part of the lateral suprasylvian sulcus (PLLS) is generally held to be an area similar to the PMLS, contributing to motion analysis, but the specific response properties for visual motion are quite different [74,119], thus, it is supposed to have different role in motion information processing. Contrary to the PMLS, which receives inputs from area 17 and other structures which also get input from the primary visual cortex (areas 18 and 19, and the lateral division of the lateral posterior nucleus) and projects to areas 17, 18, 19 and 20a and other LS areas, the PLLS receives only sparse input from striate-recipient structures, but instead it is driven mainly by tectal inputs from the medial division of the lateral posterior nucleus and projects to more remote extrastriate areas such as theAEV [74,137–140]. The reciprocal connection between the PMLS and the PLLS is weak [74,132–134]. We do not have as much as information about the PLLS as we have about the PMLS. These two regions seem to be located at roughly equivalent stages in the hierarchy but in two substreams. Li *et al*. [125] showed that the vast majority (90%) of PLLS cells respond to optic flow patterns, although only 20–25% of the neurons have been found to be selective to certain types of optic flow stimuli (*i.e.*, translation, rotation or expansion-contraction). This is consistent with the results of Kim *et al*. [141], who demonstrated that the majority of cells in the lateral suprasylvian cortex respond preferentially to optic flow movies rather than to equivalent texture movies. Additionally, Sherk *et al*. [142] have found that the PLLS neurons react mostly to objects moving against an optic flow movie rather than to a bar moving against a homogenous background. Beside this indirect evidence, direct evidence also suggests that the PLLS cortex plays an important role in figure-ground segmentation. Robitaille *et al*. [143] pursued a systematic investigation in this area to determine the spatial features of the receptive fields of the neurons, and to describe their spatial frequency tuning functions, moreover, in the second step illusory edges were created by drifting texture stimuli (*i.e.*, a horizontal bar) against a similarly textured, but static background, with the help of random-dot kinematograms. Almost all cells recorded in the PLLS (96%) were binocular, and a significant majority of the receptive fields (79.2%) were end-stopped. Most neurons (81.0%) exhibited band-pass spatial frequency tuning characteristics and reacted optimally to low spatial frequencies (mean spatial frequency: 0.08 c/deg). The remaining group of neurons (19.0%) exhibited low-pass properties. All the recorded neurons responded vigorously to edges defined by motion. The vast majority (96.0%) of neurons reacted optimally to large texture elements; approximately half the neurons (57.3%) also responded to finer texture elements. Moreover, 38.5% of the units have been found to be selective to the width of the bar. Finally, some (9%) cells responded in a transient fashion to leading and to trailing edges. In conclusion, cells in the PLLS area are low spatial frequency analyzers that are sensitive to texture and to distance between edges defined by motion.

The anteromedial lateral suprasylvian cortex (AMLS) seems to be a likely candidate for higher-order motion processing in the feline visual cortex [144–146]. Neurons in the AMLS cortex exhibit large and complex-like receptive fields, and most of them (74%) can be classified as direction-selective on the basis of their responses to drifting sinusoidal gratings [147]. Most significantly, direction selectivity was present for complex motion stimuli. A subset of the recorded neurons (21%) exhibited pattern-motion selectivity in response to moving plaid patterns. The capability of the AMLS neurons to signal higher-order stimuli was further supported by their selectivity to moving complex random-dot kinematograms. Moreover, 45% of the neurons were direction-selective when radial optic flow stimulus was applied. These results suggest that the AMLS cortex is involved in higher-order analysis of visual motion. Researches find it possible that the AMLS cortex represents a region between the PMLS and the AEV in a functional hierarchy involved in motion integration [76,147–150].

## 5. Is There a Primate Homolog of the Feline Ascending Tectofugal System?

Thorough investigations have been performed during the last few decades to explore the neuronal background of motion detection in both primates and human. In the human brain researchers found an area in the occipital cortex (V5/middle temporal area (MT)), which proved to be highly specialized for visual motion detection [151]. This area is the possible human homolog of the MT-MST area in the monkey brain, which has been in the focus of attention of researchers recently. The MT-MST area of the non-human primate brain has been proven to have anatomical connections with the areas V1, V2, V3 and V4, moreover, it has also direct input from the lateral geniculate [152,153] and pulvinar [154] nuclei of the thalamus, passing by the primary visual (striate) cortex, V1. The presence of visual fibers passing by V1 to MT provides a clear explanation for the motion sensitivity in patients after the loss of the V1 area.

Several researchers made extensive efforts to prove a direct projection from the LGN to the extrastriate cortex. A direct projection from the LGN to the MT has been reported as a result of few studies [32,152,153,155–157]. Indirect evidence for this direct thalamus-V5 connection is also available in human [158]. The PMLS area of the feline brain is generally considered to be a homolog of the MT, which is at a lower level than the MST in the visual motion pathway of the primate [34,159,160]. Previously the PLLS was regarded as a possible analog of the MST [160], but finally Li *et al*. [125] described the significantly different visual properties of the PLLS neurons (e.g., sensitive to fewer types of stimuli, the optic flow selectivity is not as good as in MST, but responds better and more selectively to radial motion), so this area cannot be considered as an equivalent of the MST.

The pulvinar nucleus of the thalamus, which was traditionally divided into 4 parts: oral (somatosensory), superior and inferior (both visual) and medial (visual and multisensory) is situated medial and dorsal to the LGN and ventral to the SC [23,161,162]. Further morphological studies defined eight to ten anatomical subdivisions [163]. However, the clear physiological properties and inter-relationships of these multiple regions are as yet unclear. It is without doubt that the majority of the pulvinar is involved in vision [23,164,165]. With their reciprocal connections to many areas of the cerebral cortex, and input from the colliculus and retina, they occupy an analogous position in the extrastriate visual system to the LGN in the primary visual pathway, but deal with higher-order visual and visuomotor transduction. The traditional view is that the inferior and lateral components are primarily associated with the striate and near-striate cortices, while elements of the lateral and the medial component are associated with higher cortices (e.g., parietal, frontal, orbital and cingulated cortices). The pathway in which the pulvinar is involved ascends from the SC and pretectum and, bypassing the LGN, reaches virtually all the visually related areas of the cerebral cortex [23].

The possibility of the existence of an SC-pulvinar-MT pathway arose, and has been investigated several times, but finally Stepniewska *et al*. [161] found that there are neurons in the medial nucleus of the inferior pulvinar—the major thalamic projection zone to the MT—that receives direct input from the SC. However, the functional role and the significance of this pathway in primates are not clear. We argue that the SC-pulvinar-MT tecto-thalamo-cortical pathway in the primate is a good candidate to be the primate homolog of the ascending tecto-thalamo (LM-Sg, LP-Pul)-cortical (AES cortex, LS areas) visual system in the feline brain.

## 6. Conclusions

Motion detection is one of the most important capabilities of the visual system. Furthermore, it provides information on our own motion. Motion is an essential image quality, which defines the visual experience regardless of other qualities, like color or contrast. Deficits of motion sensation are quite rare, however, when they occur, they disable the patient severely. In the present review we summarized the extrageniculo-extrastriate cortical and subcortical visual structures of the feline and macaque brain, and discussed their functional role in visual motion perception. Special attention was paid to the ascending tectofugal system of the feline brain that plays important roles in sensory-motor coordination and may serve for perception of the visual environment during self-motion. We also discussed the homologs, the similarities and the differences between the motion detector regions in the feline and the primate.

## Figures and Tables

**Figure 2.**
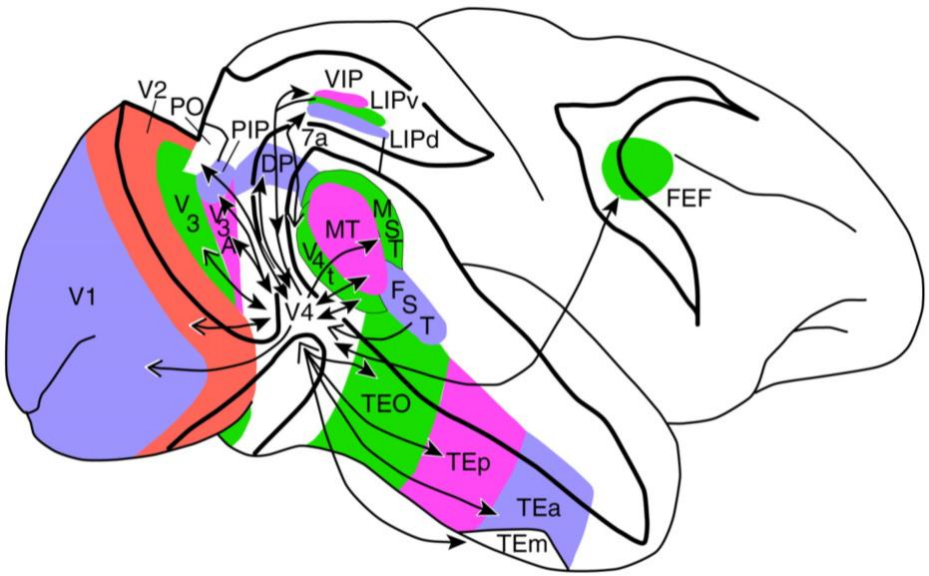
Location of the cortical visual areas in the primate. Open arrowheads indicate feedback projections, closed arrowheads indicate feedforward projections. Occipital areas: V1, V2, V3, V4—Primary (first), second, third, fourth visual cortices respectively, Temporal areas: TEO—Posterior inferior temporal cortex, TE—Anterior inferior temporal cortex, MT—Middle temporal area (fifth visual cortex), MST—Medial superior temporal area, FST—Fundus of the superior temporal area, Parietal areas: LIP—Lateral intraparietal area, VIP—Ventral intraparietal area, PIP—Posterior intraparietal area, PO—parieto-occipital sulcus, 7a—Visual area 7a in the parietal cortex (Brodmann’s terminology), Frontal area: FEF—Frontal eye field. According to the figure of Ungerleider *et al*. [73].

**Figure 3.**
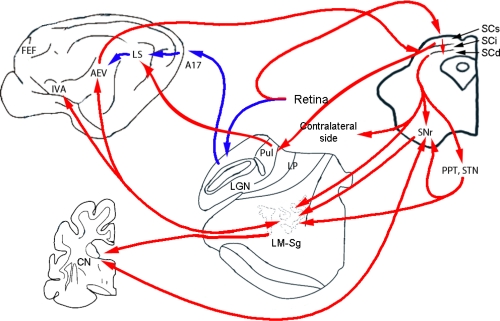
Visual pathways in the feline brain. This schematic figure shows the geniculo-cortical (primary) visual pathway (blue arrows) and the ascending tectofugal visual pathway (red arrows) in the cat’s brain. Abbreviations: LGN—Lateral geniculate nucleus, Pul—Pulvinar, LP—Lateral-posterior nucleus of the thalamus, A17—Visual area 17 (primary visual cortex), LS—Lateral suprasylvian cortex, AEV—anterior ectosylvian visual area, IVA—Insular visual area, LM-Sg—Lateral medial-suprageniculate nuclei of the thalamus, SNr—Substantia nigra pars reticulata, PPT—Pedunculo-pontin tegmental nuclei, STN—Subthalamic nucleus, SCs, SCi, SCd—Superior colliculus (superficial, intermedier, deep layers, respectively), CN—Caudate nucleus, FEF—Frontal eye field.

## References

[1] Nakayama K. (1985). Biological image motion processing: A review. Vis. Res.

[2] Braunstein M.L. (1966). Sensitivity of the observer to transformations of the visual field. J. Exp. Psychol.

[3] Simpson J.I., Leonard C.S., Soodak R.E. (1988). The accessory optic-system. Analyzer of self-motion. Ann. N. Y. Acad. Sci.

[4] Goodale M.A., Milner A.D. (1992). Separate visual pathways for perception and action. Trends Neurosci.

[5] Ungerleider L.G., Mishkin M., Ingle D.J., Goodale M.A., Mansfield R.J.W. (1982). Two cortical visual systems. Analysis of Visual Behavior.

[6] Hubel D.H., Wiesel T.N. (1962). Receptive fields, binocular interaction and functional architecture in the cat’s visual cortex. J. Physiol. Lond.

[7] Hubel D.H., Wiesel T.N. (1968). Receptive fields and functional architecture of monkey striate cortex. J. Physiol. Lond.

[8] Snowden R.J., Treue T., Erickson R.G., Anderson R.A. (1991). The response of area Mt and V1 neurons to transparent motion. J. Neurosci.

[9] Movshon J.A., Newsome W.T. (1996). Visual response properties of striate cortical neurons projecting to area MT in macaque monkey. J. Neurosci.

[10] Clifford C.W.G., Ibbotson M.R. (2002). Fundamental mechanisms of visual motion detection: models, cells and functions. Prog. Neurobiol.

[11] Andersen R.A. (1997). Neural mechanisms of visual motion perception in primates. Neuron.

[12] Snowden R.J., Freeman T.C.A. (2004). The visual perception of motion. Curr. Biol.

[13] Adelson E.H., Movshon J.A. (1982). Phenomenal coherence of moving visual patterns. Nature.

[14] Welch L. (1989). The perception of moving plaids reveals two motion-processing stages. Nature.

[15] Derrington A, Suero M. (1991). Motion of complex patterns is computed from the perceived motions of their components. Vision Res.

[16] Albright T.D., Stoner G.R. (1995). Visual motion perception. Proc. Natl. Acad. Sci. USA.

[17] Li B., Chen Y., Li B.W., Wang L.H., Diao Y.C. (2001). Pattern and component motion selectivity in cortical area PMLS of the cat. Eur. J. Neurosci.

[18] Dacey D.M., Peterson B.B., Robinson F.R., Gamlin P.D. (2003). Fireworks in the primate retina: *In vitro* photodynamics reveals diverse LGN-projecting ganglion cell types. Neuron.

[19] Hendry S.H., Reid R.C. (2000). The koniocellular pathway in primate vision. Annu. Rev. Neurosci.

[20] Kuffler S. (1953). Discharge patterns and functional organization of the mammalian retina. J. Neurophysiol.

[21] Croner L.J., Kaplan E. (1995). Receptive fields of P and M ganglion cells across the primate retina. Vision Res.

[22] Barlow H.B., Levick W.R. (1965). The mechanism of directionally selective units in rabbit’s retina. J. Physiol.

[23] Jones E.G. (1985). The Thalamus.

[24] Kaplan E., Shapley R.M. (1982). X and Y cells in the lateral geniculate nucleus of macaque monkeys. J. Physiol.

[25] Cavanagh P., Tyler C.W., Favreau O.E. (1984). Perceived velocity of moving chromatic gratings. J. Opt. Soc. Am. A.

[26] Ramachandran V.S., Gregory R.L. (1978). Does color provide an input to human motion perception?. Nature.

[27] Gur M., Snodderly D.M. (2007). Direction selectivity in V1 of alert monkeys: evidence for parallel pathways for motion processing. J Physiol.

[28] Livingstone M.S., Hubel D.H. (1988). Do the relative mapping densities of the magno- and parvocellular systems vary with eccentricity?. J. Neurosci.

[29] Shipp S., Zeki S. (1989). The Organization of Connections between Areas V5 and V1 in Macaque Monkey Visual Cortex. Eur. J. Neurosci.

[30] Nassi J.J., Callaway E.M. (2007). Specialized circuits from primary visual cortex to V2 and area MT. Neuron.

[31] Felleman D.J., Burkhalter A., Van Essen D.C. (1997). Cortical connections of areas V3 and VP of macaque monkey extrastriate visual cortex. J. Comp. Neurol.

[32] Nassi J.J., Callaway E.M. (2009). Parallel processing strategies of the primate visual system. Nat. Rev. Neurosci.

[33] Allman J.M., Kaas J.H. (1974). A crescent-shaped cortical visual area surrounding the middle temporal area (MT) in the owl monkey (Aotus trivirgatus). Brain Res.

[34] Zeki S.M. (1974). Functional organization of a visual area in the posterior bank of the superior temporal sulcus of the rhesus monkey. J. Physiol.

[35] Van Essen D.C. (1979). Visual areas of the mammalian cerebral cortex. Ann. Rev. Neurosci.

[36] Zeki S.M. (1971). Convergent input from the striate cortex (area 17) to the cortex of the superior temporal sulcus in the rhesus monkey. Brain Res.

[37] Zeki S.M. (1971). Cortical projections from two prestriate areas in the monkey. Brain Res.

[38] Maunsell J.H., Van Essen D.C. (1983). Functional properties of neurons in middle temporal visual area of the macaque monkey. I. Selectivity for stimulus direction, speed, and orientation. J. Neurophysiol.

[39] Albright T.D., Desimone R., Gross C.G. (1984). Columnar organization of directionally selective cells in visual area MT of the macaque. J. Neurophysiol.

[40] Newsome W.T., Wurtz R.H., Dürsteler M. R., Mikami A. (1985). Deficits in visual motion processing following ibotenic acid lesions of the middle temporal visual area of the macaque monkey. J. Neurosci.

[41] Rashbass C. (1961). The relationship between saccadic and smooth tracking eye movements. J. Physiol.

[42] Suzuki D.A., Noda H., Kase M. (1981). Visual and pursuit eye movement-related activity in posterior vermis of monkey cerebellum. J. Neurophysiol.

[43] Suzuki D.A., Keller E.L. (1984). Visual signals in the dorsolateral pontine nucleus of the alert monkey: their relationship to smooth-pursuit eye movements. Exp. Brain Res.

[44] Movshon J.A., Newsome W.T. (1984). Functional characteristics of striate cortical neurons projecting to MT in the macaque. Soc. Neurosci. Abstr.

[45] Bradley D.C., Chang G.C., Andersen R.A. (1998). Encoding of three-dimensional structure-from-motion by primate area MT neurons. Nature.

[46] Grunewald A., Bradley D.C., Andersen R.A. (2002). Neural correlates of structure-from-motion perception in macaque V1 and MT. J. Neurosci.

[47] Shipp S., Zeki S. (1989). The Organization of Connections between Areas V5 and V2 in Macaque Monkey Visual Cortex. Eur. J. Neurosci.

[48] Vaina L.M., Cowey A., Eskew R.T., LeMay M., Kemper T. (2001). Regional cerebral correlates of global motion perception: evidence from unilateral cerebral brain damage. Brain.

[49] Perge A.J., Borghuis B.G., Bours R.J.E., Lankheet M.J.M., van Wezel R.J.A. (2005). Temporal dynamics of direction tuning in motion-sensitive macaque area MT. J. Neurophysiol.

[50] Serences J.T., Boynton G.M. (2007). The representation of behavioral choice for motion in human visual cortex. J. Neurosci.

[51] Zeki S., Watson J.D.G., Frackowiak R.S.J. (1993). Going beyond the information given: the relation of illusory visual motion to brain activity. Proc. Biol. Sci.

[52] Williford T., Maunsell J.H. (2006). Effects of spatial attention on contrast response functions in macaque area V4. J. Neurophysiol.

[53] Martínez-Trujillo J., Treue S. (2002). Attentional modulation strength in cortical area MT depends on stimulus contrast. Neuron.

[54] Patzwahl D.R., Treue S. (2009). Combining spatial and feature-based attention within the receptive field of MT neurons. Vision Res.

[55] Treue S. (2001). Neural correlates of attention in primate visual cortex. Trends Neurosci.

[56] Lui L.L., Bourne J.A., Rosa M.G.P. (2007). Spatial and temporal frequency selectivity of neurons in the middle temporal visual area of new world monkeys (Callithrix jacchus). Eur. J. Neurosci.

[57] Ungerleider L.G., Desimone R.J. (1986). Cortical connections of visual area MT in the macaque. Comp. Neurol.

[58] Maunsell J.H., van Essen D.C. (1983). The connections of the middle temporal visual area (MT) and their relationship to a cortical hierarchy in the macaque monkey. J. Neurosci.

[59] Eifuku S., Wurtz R.H. (1998). Response to motion in extrastriate area MSTl: center-surround interactions. J. Neurophysiol.

[60] Britten K.H., Van Wezel R.J. (2002). Area MST and heading perception in macaque monkeys. Cereb. Cortex.

[61] Rizzolatti G., Fogassi L., Gallese V. (1997). Parietal cortex: from sight to action. Curr. Opin. Neurobiol.

[62] Kalaska J.F., Scott S.H., Cisek P., Sergio L.E. (1997). Cortical control of reaching movements. Curr. Opin. Neurobiol.

[63] Culham J.C., Cavina-Pratesi C., Singhal A. (2006). The role of parietal cortex in visuomotor control: what have we learned from neuroimaging?. Neuropsychologia.

[64] Sakata H., Taira M., Murata A., Mine S. (1995). Neural Mechanisms of visual guidance of hand action in the parietal cortex of monkey. Cereb. Cortex.

[65] Sakata H., Taira M., Kusunoki M., Murata A., Tanaka Y. (1997). The TINS lecture. The parietal association cortex in depth perception and visual control of hand action. Trends Neurosci.

[66] Motter B.C., Mountcastle V.B. (1981). The functional properties of the light-sensitive neurons of the posterior parietal cortex studied in waking monkeys: foveal sparing and opponent vector organization. J. Neurosci.

[67] Collewijn H., Curio G., Grüsser O.J. (1982). Spatially selective visual attention and generation of eye pursuit movements. Experiments with sigma-movement. Hum. Neurobiol.

[68] Carman J.B., Cowan W.M., Powell T.P., Webster K.E. (1965). A bilateral cortico-striate projection. J. Neurol. Neurosurg. Psychiatry.

[69] Atkinson J., Freeman R.D. (1979). Development of optokinetic nystagmus in the human infant and monkey infant: an analogue to development in kittens. Developmental Neurobiology of Vision.

[70] Atkinson J., Braddick O.J., Aslin R.N., Alberts J.R., Petersen M.R. (1981). Acuity, contrast sensitivity and accommodation in infancy. The Development of Perception.

[71] Croner L.J., Albright T.D. (1999). Seeing the big picture: integration of image cues in the primate visual system. Neuron.

[72] Albright T.D., Stoner G.R. (1995). Visual motion perception. Proc. Natl. Acad. Sci. USA.

[73] Ungerleider L.G., Galkin T.W., Desimone R., Gattass R. (2008). Cortical connections of area V4 in the macaque. Cereb. Cortex.

[74] Rosenquist A.C., Peters A., Jones E.G. (1985). Connections of visual Cortical Areas in the Cat. Cerebral Cortex.

[75] Mucke L., Norita M., Benedek G., Creutzfeldt O. (1982). Physiologic and anatomic investigation of a visual cortical area situated in the ventral bank of the anterior ectosylvian sulcus of the cat. Exp. Brain Res.

[76] Olson C.R., Graybiel A.M. (1987). Ectosylvian visual area of the cat: location, retinotopic organization, and connections. J. Comp. Neurol.

[77] Olson C.R., Graybiel A.M. (1983). An outlying visual area in the cerebral cortex of the cat. Prog. Brain Res.

[78] Benedek G., Jang E.K., Hicks T.P. (1986). Physiological properties of visually responsive neurons in the insular cortex of the cat. Neurosci. Lett.

[79] Hicks T.P., Benedek G., Thurlow G.A. (1988). Organization and properties of neurons in a visual area within the insular cortex of the cat. J. Neurophysiol.

[80] Norita M., Hicks T.P., Benedek G., Katoh Y.Y. (1991). Organization of cortical and subcortical projections to the feline insular visual area, IVA. J. Hirnforsch.

[81] Reinoso-Suarez F., Roda J.M. (1985). Topographical organization of the cortical afferent connections to the cortex of the anterior ectosylvian sulcus in the cat. Exp. Brain Res.

[82] Hoshino K., Horie M., Nagy A., Berényi A., Benedek G., Norita M. (2009). Direct synaptic connections between superior colliculus afferents and thalamo-insular projection neurons in the feline suprageniculate nucleus: A double-labeling study with WGA-HRP and kainic acid. Neurosci. Res.

[83] Norita M., Mucke L., Benedek G., Albowitz B., Katoh Y.Y., Creutzfeldt O.D. (1986). Connections of the anterior ectosylvian visual area (AEV). Exp. Brain Res.

[84] Miceli D., Reperant J., Ptito M. (1985). Intracortical connections of the anterior ectosylvian and lateral suprasylvian visual areas in the cat. Brain Res.

[85] Hardy H., Heimer L., Switzer R., Watkins D. (1976). Simultaneous demonstration of horseradish peroxydase and acetylcholinesterase. Neurosci. Lett.

[86] Katoh Y.Y., Benedek G. (1995). Organization of the colliculo-suprageniculate pathway in the cat: a wheat germ agglutinin-horseradish peroxydase study. J. Comp. Neurol.

[87] Katoh Y.Y., Benedek G., Deura S. (1995). Bilateral projections from the superior colliculus to the suprageniculate nucleus in the cat: a WGA-HRP/double fluorescent tracing study. Brain Res.

[88] Crapse T.B., Sommer M.A. (2009). Frontal eye field neurons with spatial representations predicted by their subcortical input. J. Neurosci.

[89] Hoshino K., Eördegh G., Nagy A., Benedek G., Norita M. (2009). Overlap of nigrothalamic terminals and thalamostriatal neurons in the feline lateralis medialis-suprageniculate nucleus. Acta Physiol. Hung.

[90] Katoh Y.Y., Arai R., Benedek G. (2000). Bifurcating projections from the cerebellar fastigial neurons to the thalamic suprageniculate nucleus and to the superior colliculus. Brain Res.

[91] Guirado S., Real M.A., Dávila J.C. (2005). The ascending tectofugal visual system in amniotes: New insights. Brain Res. Bull.

[92] Rokszin A., Márkus Z., Braunitzer G., Berényi A., Wypych M., Waleszczyk W.J., Benedek G., Nagy A. (2009). Spatio-temporal visual properties in the ascending tectofugal system. Cent. Eur. J. Biol.

[93] Benedek G., Hicks T.P. (1988). The visual insular cortex of the cat: organization, properties and modality specificity. Prog. Brain Res.

[94] Benedek G., Perény J., Kovács G., Fischer-Szatmári L., Katoh Y.Y. (1997). Visual, somatosensory, auditory and nociceptive modality properties in the feline suprageniculate nucleus. Neuroscience.

[95] Tusa R.J., Palmer L.A., Rosenquist A.C. (1978). The retinotopic organization of area 17 (striate cortex) in the cat. J. Comp. Neurol.

[96] Scannell J.W., Sengpiel F., Tovee M.J., Benson P.J., Blakemore C., Young M.P. (1996). Visual motion processing in the anterior ectosylvian sulcus of the cat. J. Neurophysiol.

[97] Benedek G., Mucke L., Norita M., Albowitz B., Creutzfeldt O.D. (1988). Anterior ectosylvian visual area (AEV) of the cat: physiological properties. Prog. Brain Res.

[98] Middlebrooks J.C., Clock A.E., Xu L., Green D.M. (1994). A panoramic code for sound location by cortical neurons. Science.

[99] Benedek G., Sztriha L., Kovács G. (2000). Coding of spatial co-ordinates on neurons of the feline visual association cortex. Neuroreport.

[100] Stein B.E., Meredith M.A. (1993). The Merging of the Sense.

[101] De Valois K.K., De Valois R.L., Yund E.W. (1979). Responses of striate cortex cells to grating and checkerboard patterns. J. Physiol.

[102] Pinter R.B., Harris L.R. (1981). Temporal and spatial response characteristics of the cat superior colliculus. Brain Res.

[103] Enroth-Cugell C., Robson J.G. (1966). The contrast sensitivity of retinal ganglion cells of the cat. J. Physiol.

[104] Zumbroich T., Price D.J., Blakemore C. (1988). Development of spatial and temporal selectivity in the suprasylvian visual cortex of the cat. J. Neurosci.

[105] Anderson S.J., Burr D.C. (1985). Spatial and temporal selectivity of the human motion detection system. Vision Res.

[106] Burr D.C., Ross J. (1982). Contrast sensitivity at high velocities. Vision Res.

[107] Burr D.C., Morrone M.C., Ross J. (1986). Local and global visual processing. Vision Res.

[108] Morrone M.C., Di Stefano M., Burr D.C. (1986). Spatial and temporal properties of neurons of the lateral suprasylvian cortex of the cat. J. Neurophysiol.

[109] Brosseau-Lachaine O., Faubert J., Casanova C. (2001). Functional subregions for optic flow processing in the posteromedial lateral suprasylvian cortex of the cat. Cereb. Cortex.

[110] Palmer L.A., Rosenquist A.C., Tusa R.J. (1978). The retinotopic organization of lateral suprasylvian visual areas in the cat. J. Comp. Neurol.

[111] Ogashawara K., McHaffie J.G., Stein B.E. (1984). Two visual corticotectal systems in cat. J. Neurophysiol.

[112] Hardy S.C., Stein B.E. (1988). Small lateral suprasylvian cortex lesion produce visual neglect and decreased visual activity in the superior colliculus. J. Comp. Neurol.

[113] Payne B.R., Lomber S.G., Geeraerts S., van der Gucht E., Vandenbusschen E. (1996). Reversible visual hemineglect. Proc. Natl. Acad. Sci. USA.

[114] Pasternak T., Horn K.M., Maunsell J.H. (1989). Deficits in speed discrimination following lesions of the lateral suprasylvian cortex in the cat. Vis. Nerosci.

[115] Rudolph K.K., Pasternak T. (1996). Lesions in cat lateral suprasylvian cortex affect the perception of complex motion. Cereb. Cortex.

[116] Kiefer W., Kruger K., Strauss G., Berlucchi G. (1989). Considerable deficits in the detection performance of the cat after lesion of the suprasylvian visual cortex. Exp. Brain Res.

[117] Krüger K., Kiefer W., Groh A., Dinse H.R., von Seelen W. (1993). The role of the lateral suprasylvian cortex of the cat in object-background interactions: permanent deficits following lesions. Exp. Brain Res.

[118] Spear P.D., Baumann T.P. (1975). Receptive-field characteristics of single neurons in lateral suprasylvian visual area of the cat. J. Neurophysiol.

[119] Blakemore C., Zumbroich T.J. (1987). Stimulus selectivity and functional organization in the lateral suprasylvian visual cortex of the cat. J. Physiol. (London).

[120] Rauschecker J.P., von Grünau M.W., Poulin C. (1987). Centrifugal organization of direction preferences in the cat’s lateral suprasylvian visual cortex and its relation to flow field processing. J. Neurosci.

[121] von Grünau M.W., Zumbroich T.J., Poulin C. (1987). Visual receptive field properties in the posterior suprasylvian cortex of the cat: a comparison between areas PMLS and PLLS. Vision Res.

[122] Gizzi M.S., Katz E., Movshon J.A. (1990). Spatial and temporal analysis by neurons in the representation of the central visual field in the cat’s lateral suprasylvian visual cortex. Vis. Neurosci.

[123] Gizzi M.S., Katz E., Schumer R.A., Movshon J.A. (1990). Selectivity for orientation and direction of motion of single neurons in cat striate and extrastriate visual cortex. J. Neurophysiol.

[124] Minville K., Casanova C. (1998). Spatial frequency processing in the posteromedial lateral suprasylvian cortex does not depend on the projections from the striate-recipient zone of the cat’s lateral posterior-pulvinar complex. Neurosciences.

[125] Li B., Li B.W., Chen Y., Wang L.H., Diao Y.C. (2000). Response properties of PMLS and PLLS neurons to stimulated optic flow patterns. J. Neurosci.

[126] Brosseau-Lachaine O., Faubert J., Casanova C. (2001). Functional sub-regions for optic flow processing in the posteromedial lateral suprasylvian cortex of the cat. Cereb. Cortex.

[127] Morrone M.C., Di Stefano M., Burr D.C. (1986). Spatial and temporal properties of neurons of the lateral suprasylvian cortex of the cat. J. Neurophysiol.

[128] von Grünau M., Frost B.J. (1983). Double-opponent-process mechanism underlying RF-structure of directionally specific cells of cat lateral suprasylvian visual area. Exp. Brain Res.

[129] Yin T.C., Greenwood M. (1992). Visuomotor interactions in responses of neurons in the middle and lateral suprasylvian cortices of the behaving cat. Exp. Brain Res.

[130] Sherk H., Fowler G.A. (2002). Lesions of extrastriate cortex and consequences for visual guidance during locomotion. Exp. Brain Res.

[131] Camarda R., Rizzolatti G. (1976). Visual receptive fields in the lateral suprasylvian area (Clare-Bishop area) of the cat. Brain Res.

[132] Toyama K., Mizobe K., Akase E., Kaihara T. (1994). Neuronal responsiveness in areas 19 and 21a, and the posteromedial lateral suprasylvian cortex of the cat. Exp. Brain Res.

[133] Hamada T. (1987). Neural response to the motion of textures in the lateral suprasylvian area of cats. Behav. Brain Res.

[134] Danilov Y., Moore R.J., King V.R., Spear P.D. (1995). Are neurons in cat posteromedial lateral suprasylvian visual cortex orientation sensitive? Tests with bars and gratings. Vis. Neurosci.

[135] Dreher B., Wang C., Turlejski K.J., Djavadian R.L., Burke W. (1996). Areas PMLS and 21a of cat visual cortex: two functionally distinct areas. Cereb. Cortex.

[136] Villeneuve M.Y., Ptito M., Casanova C. (2006). Global motion integration in the postero-medial part of the lateral suprasylvian cortex in the cat. Exp. Bran Res.

[137] Dreher B., Pettigrew J.D., Sanderson K.J., Levick W.R. (1986). Thalamocortical and corticocortical interconnections in the cat visual system: relation to the mechanisms of information processing. Visual Neuroscience.

[138] Sherk H. (1986). Location and connections of visual cortical areas in the cat’s suprasylvian sulcus. J. Comp. Neurol.

[139] Grant S., Shipp S. (1991). Visuotopic organization of the lateral suprasylvian area and of an adjacent area of the ectosylvian gyrus of cat cortex: A physiological and connectional study. Vis. Neurosci.

[140] Scannell J.W., Blakemore C., Young M.P. (1995). Analysis of connectivity in the cat cerebral cortex. J. Neurosci.

[141] Kim J.N., Mulligan K., Sherk H. (1997). Simulated optic flow and extrastriate cortex. I. Optic flow versus texture. J. Neurophysiol.

[142] Sherk H., Mulligan K., Kim J.N. (1997). Neuronal responses in extrastriate cortex to objects in optic flow fields. Vis. Neurosci.

[143] Robitaille N., Lepore F, Bacon B.A., Ellemberg D., Guillemot J.P. (2008). Receptive field properties and sensitivity to edges defined by motion in the postero-lateral lateral suprasylvian (PLLS) area of the cat. Brain Res.

[144] Lomber S.G. (2001). Behavioral cartography of visual functions in cat parietal cortex: areal and laminar dissociations. Prog. Brain Res.

[145] Vanduffel W., Vandenbusschen E., Singer W., Orban G.A. (1997). A metabolic study of orientation discrimination and detection tasks in the cat. Eur. J. Neurol.

[146] Sprague J.M., De Weerd P., Xiao D.K., Vandenbusschen E., Orban G.A. (1996). Orientation discrimination in the cat: its cortical locus: II. Extrastriate cortical areas. J. Comp. Neurol.

[147] Ouellette B.G., Minville K., Faubert J., Casanova C. (2004). Simple and complex visual motion response properties in the anterior medial bank of the lateral suprasylvian cortex. Neuroscience.

[148] Symonds L.L., Rosenquist A.C. (1984). Corticocortical connections among visual areas in the cat. J. Comp. Neurol.

[149] Symonds L.L., Rosenquist A.C. (1984). Laminar origins of visual corticocortical connections in the cat. J. Comp. Neurol.

[150] Miceli D., Repérant J., Ptito M. (1985). Intracortical connections of the anterior ectosylvian and lateral suprasylvian visual areas in the cat. Brain Res.

[151] Zeki S., Watson J.D., Lueck C.J., Friston K.J., Kennard C., Frackowiak R.S. (1991). A direct demonstration of functional specialization in human cortex. J. Neurosci.

[152] Fries W. (1981). The projection from the lateral geniculate nucleus to the peristriate cortex of the macaque monkey. Proc. R. Soc. Lond. B. Biol.

[153] Yukie M., Iwai E. (1981). Direct projection from the dorsal lateral geniculate nucleus to the peristriate cortex in macaque monkey. J. Comp. Neurol.

[154] Standage G.P., Benevento L.A. (1983). The organization of connections between the pulvinar and visual area MT in the macaque monkey. Brain Res.

[155] Gross C.G. (1991). Contribution of striate cortex and the superior colliculus to visual function in area MT, the superior temporal polysensory area and the inferior temporal cortex. Neuropsychologia.

[156] Rodman H.R., Gross C.G., Albright T.D. (1989). Afferent basis of visual response properties in area MT of the macaque. I. Effects of striate cortex removal. J. Neurosci.

[157] Rodman H.R., Gross C.G., Albright T.D. (1990). Afferent basis of visual response properties in area MT of the macaque. II. Effects of superior colliculus removal. J. Neurosci.

[158] Schoenfeld M.A., Heinze H.-J., Woldorff M.G. (2002). Unmasking motion-processing activity in human brain area V5/MT+ mediated by pathways that bypass primary visual cortex. Neuroimage.

[159] Felleman D.J., Van Essen D.C. (1991). Distributed hierarchical processing in the primate cerebral cortex. Cereb. Cortex.

[160] Payne B.R. (1993). Evidence for visual cortical area homologs in cat and macaque monkey. Cereb. Cortex.

[161] Stepniewska I., Qi H.X., Kaas J.H. (1999). Do superior colliculus projection zones in the inferior pulvinar project to MT in primates?. Eur. J. Neurosci.

[162] Gutierrez C., Cusick C.G. (1997). Area V1 in macaque monkeys projects to multiple histochemically defined subdivisions of the inferior pulvinar complex. Brain Res.

[163] Cavada C., Compañy T., Hernández-González A., Reinoso-Suárez F. (1995). Acetylcholinesterase histochemistry in the macaque thalamus reveals territories selectively connected to frontal, parietal and temporal association cortices. J. Chem. Neuroanat.

[164] Robinson D.L. (1993). Functional contributions of the primate pulvinar. Prog. Brain Res.

[165] Grieve K.L., Acuña C., Cudeiro J. (2000). The primate pulvinar nuclei: vision and action. Trends Neurosci.

